# Associations Between Exposure to and Expression of Negative Opinions About Human Papillomavirus Vaccines on Social Media: An Observational Study

**DOI:** 10.2196/jmir.4343

**Published:** 2015-06-10

**Authors:** Adam G Dunn, Julie Leask, Xujuan Zhou, Kenneth D Mandl, Enrico Coiera

**Affiliations:** ^1^Centre for Health InformaticsAustralian Institute of Health InnovationMacquarie UniversitySydneyAustralia; ^2^School of Public HealthThe University of SydneySydneyAustralia; ^3^Children’s Hospital Informatics ProgramBoston Children’s HospitalBoston, MAUnited States; ^4^Center for Biomedical InformaticsHarvard Medical SchoolBoston, MAUnited States

**Keywords:** HPV vaccines, Twitter messaging, social media, public health surveillance, social networks

## Abstract

**Background:**

Groups and individuals that seek to negatively influence public opinion about the safety and value of vaccination are active in online and social media and may influence decision making within some communities.

**Objective:**

We sought to measure whether exposure to negative opinions about human papillomavirus (HPV) vaccines in Twitter communities is associated with the subsequent expression of negative opinions by explicitly measuring potential information exposure over the social structure of Twitter communities.

**Methods:**

We hypothesized that prior exposure to opinions rejecting the safety or value of HPV vaccines would be associated with an increased risk of posting similar opinions and tested this hypothesis by analyzing temporal sequences of messages posted on Twitter (tweets). The study design was a retrospective analysis of tweets related to HPV vaccines and the social connections between users. Between October 2013 and April 2014, we collected 83,551 English-language tweets that included terms related to HPV vaccines and the 957,865 social connections among 30,621 users posting or reposting the tweets. Tweets were classified as expressing negative or neutral/positive opinions using a machine learning classifier previously trained on a manually labeled sample.

**Results:**

During the 6-month period, 25.13% (20,994/83,551) of tweets were classified as negative; among the 30,621 users that tweeted about HPV vaccines, 9046 (29.54%) were exposed to a majority of negative tweets. The likelihood of a user posting a negative tweet after exposure to a majority of negative opinions was 37.78% (2780/7361) compared to 10.92% (1234/11,296) for users who were exposed to a majority of positive and neutral tweets corresponding to a relative risk of 3.46 (95% CI 3.25-3.67, *P*<.001).

**Conclusions:**

The heterogeneous community structure on Twitter appears to skew the information to which users are exposed in relation to HPV vaccines. We found that among users that tweeted about HPV vaccines, those who were more often exposed to negative opinions were more likely to subsequently post negative opinions. Although this research may be useful for identifying individuals and groups currently at risk of disproportionate exposure to misinformation about HPV vaccines, there is a clear need for studies capable of determining the factors that affect the formation and adoption of beliefs about public health interventions.

## Introduction

In the last decade, vaccination refusal has increased in the United States and many countries have recorded substantial proportions of parents expressing concerns about the safety of vaccines [1,2]. Although variability in access to health care is an important factor influencing vaccine coverage rates, vaccination refusal also directly affects these rates and is a significant contributor to outbreaks—especially where vaccination refusal is geographically clustered and population immunity is compromised [3]. Outbreaks of pertussis and measles are known to spread through populations where rates of vaccination refusal are high [4-7].

Refusal has also been a problem for the recently introduced human papillomavirus (HPV) vaccine. The vaccine was first licensed for use in the United States in 2006 with the purpose of reducing the incidence of HPV, to which the majority of cervical cancers are attributed, as well as genital warts and some oral, anal, and penile cancers [8]. HPV vaccination in Australia has led to a marked reduction in rates of high-grade cervical abnormalities and early evidence of herd immunity [9-12]. However, uptake of HPV vaccines varies substantially across and within countries [13-16].

The introduction of HPV vaccination was hampered by controversy in some countries, where some parents attributed illness or death in their children to the vaccine despite evidence affirming the vaccine’s good safety record [17]. The quality and variety of information available online about the safety and efficacy of HPV vaccines varies [18], as does the representation of HPV vaccines in the news media [19]. Evidence from a study set in Greece suggests that the perception of risks in the community appears to have negatively influenced the intention to vaccinate [20]. More generally, there is some evidence to suggest that influence from online media and celebrities can increase vaccine risk perception and rates of vaccination refusal [21-23]. Given the importance of information sources in influencing vaccination decision making, social media platforms are seen as an opportunity for both the tracking and influencing of vaccination decision making [24].

Few studies have considered the surveillance of opinions about vaccination on social media as a precursor to vaccination decision making. Existing studies on public health surveillance applications in social media have focused primarily on finding early indicators of infectious diseases incidence [25-28]. The exceptions include examinations of responses to an influenza outbreak [29] and influenza vaccination [30]. Beyond social media, media surveillance systems have been built to track news media and other reports online [31,32]. One example considered negative sentiment in online news media and notes that systems that rely on manual classification of documents are prohibitively resource intensive [33].

Our aim was to examine the association between exposure to negative opinions about HPV vaccines and the expression of negative opinions about HPV vaccines among Twitter users. To do this, we examined sequences of messages posted on Twitter (tweets) as well as a static view of the social connections between every user that posted a tweet about HPV vaccines in a 6-month period.

## Methods

### Data

Tweets posted by public users were retrieved programmatically via the Application Programming Interface (API) using repeated searches of combinations of the terms human papillomavirus, HPV, vaccine, vaccination, Gardasil, and Cervarix, and labeled by Twitter as English language. These terms were fixed throughout the data collection period, which was from October 1, 2013 to April 1, 2014. We additionally collected metadata associated with the tweets, including the date and time, information about the user, related tweets such as retweets and replies, and the geo-tag (location) information if it was available. For each user who posted one or more tweets about HPV vaccines in the period, we separately used the API to retrieve the lists of users they followed and the users that followed them shortly after the first time they posted a tweet about HPV vaccines during the period.

Tweets were classified as negative if they rejected the safety or value of HPV vaccines or promoted refusal. Due to the very large number of tweets collected in the period, we used a supervised machine learning approach to classify the tweets that involved the manual labeling of a random sample of tweets, which were then used to train algorithms that recognized similar patterns in the remaining tweets. For each tweet, we determined an estimate of the likelihood of it being the expression of a negative opinion about HPV vaccines. The specific classifier we constructed was an ensemble of 4 classifiers that used the content of the tweets (the words and word combinations in the tweets themselves) or the social relations between users (the users followed by the user responsible for the tweet). A set of 2098 tweets were randomly sampled and then independently graded by 2 investigators (95% agreement, Cohen’s κ=.87), with disagreements resolved by discussion to produce the final training set. The accuracies of the 4 machine learning classifiers ranged between 87.6% and 94.0% when trained and tested in a 10-fold cross validation. The complete details of the development of the classifier are described elsewhere [34].

### Analyses

To analyze population-level information exposure, we measured how users may have been exposed to tweets about HPV vaccines during the 6-month observation period. For each user that tweeted at least once about HPV vaccines during the period, we created timelines of their own tweets about HPV vaccines and the tweets about HPV vaccines posted by the users they followed. For the purpose of measuring information exposure, we handled retweets in the same way as other tweets to conserve the definition for exposure. This means that we defined an exposure as the potential flow of information between users along social connections. Not all tweets are seen by all followers, but by observing the aggregate flow of exposures through network structure, it was possible to estimate how the heterogeneous mixing of the population might affect the information to which each user is exposed.

We determined the prior exposure of a user each time they posted a tweet about HPV vaccines during the time period by compiling the list of tweets to which they were potentially exposed prior to the timestamp of the index tweet. This proportion served as an indicator of the prior exposure to negative information about HPV vaccines in the time period. To account for a potential length sampling bias (later tweets tended to be preceded by a greater number of exposures), we limited the sequence-based analysis to tweets that were preceded by at least 3 exposures.

To test our hypothesis directly, we counted how many times a user posted a negative tweet following a majority of prior negative exposures and compared that count with the number of times a posted tweet was negative when the majority of prior exposures were neutral or positive. These counts were then used to calculate the relative risk of posting a negative tweet about HPV vaccines given majority prior exposure to negative tweets. To avoid sampling biases resulting from counting the same users repeatedly, we randomly sampled only 1 tweet from each eligible user and repeated the analysis until the median proportions and relative risk measures did not change value at 3 significant figures.

## Results

**Figure 1:**
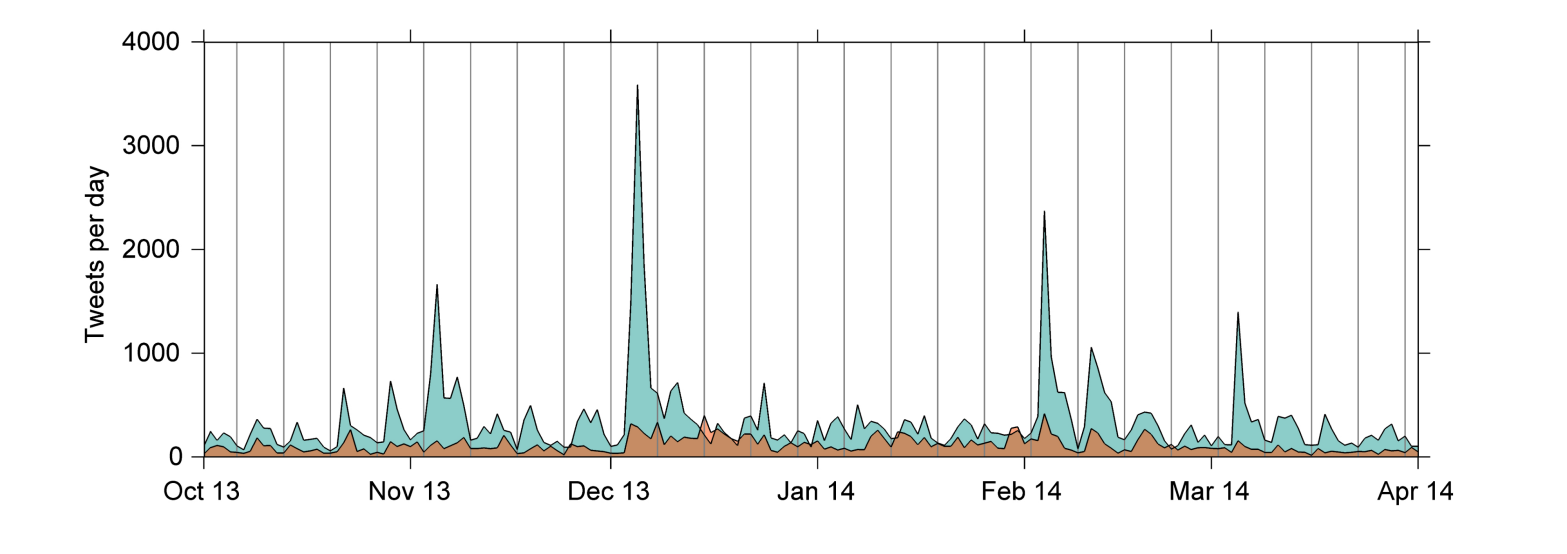
The number of tweets posted each day during the data collection period, including tweets rejecting the safety or value of HPV vaccines (orange) and all other HPV vaccine tweets (cyan). Gray vertical lines indicate Sundays. No corrections for time zone differences were applied.

We identified 83,551 tweets or retweets from 30,621 users relating to HPV vaccines between the period October 1, 2013 to April 1, 2014, after eliminating tweets that were eventually deleted and tweets from users that became protected or suspended after the initial collection. Of the 83,551 tweets and retweets, 20,994 (25.13%) were classified as negative by an ensemble of supervised machine learning classifiers. Table 1 includes some examples of the different classes of tweets. There were 10 days (5.5% of 183 days) in which the number of negative tweets outnumbered the number of positive and neutral tweets about HPV vaccines (Figure 1).

**Table 1:** Examples of different classes of Twitter messages identified in the searches.

Classification	Twitter message text
Positive	“HPV vaccination has the potential to reduce cervical cancer deaths worldwide by as much as two-thirds. [URL removed]”
Positive	“Oral sex & male gender indep assoc with oral HPV infection: shows need for HPV vaccination of boys. #endhpv New study [URL removed]”
Neutral	“Potential of the quadrivalent human papillomavirus vaccine in the prevention and treatment of cervical cancer [URL removed]”
Negative	“Gardasil has generated nearly 30,000 adverse reaction reports to US govt, including 140 deaths [URL removed] #vaxfax”
Negative	“Lead Developer of HPV Vaccine Warns Parents Young Girls It’s a Giant Deadly Scam [URL removed]”
Negative	“Young woman’s ovaries destroyed by Gardasil: Merck ‘forgot to research’ effects of vaccine [URL removed]”

**Figure 2:**
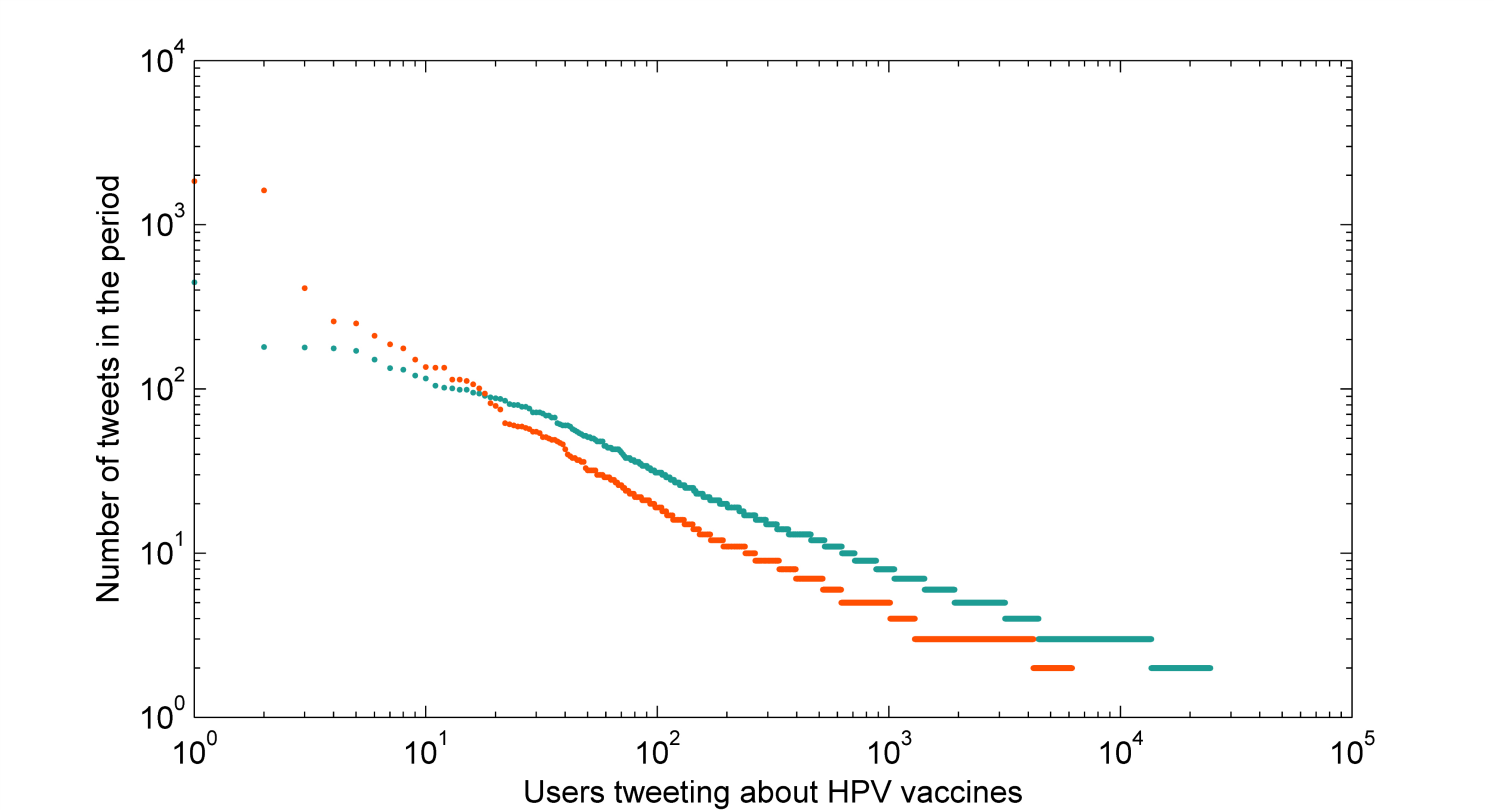
The ordered distribution of tweets per user related to HPV vaccines posted to Twitter between October 1, 2013 and March 31, 2014. Each user’s number of tweets is represented by a dot and illustrated separately for users that posted a majority of negative tweets (orange) and all other users (cyan).

There were 30,621 users that tweeted about HPV vaccines in the period. Each user in the set posted between 1 and 1842 tweets about HPV vaccines during the period with a median of 2 tweets per user (IQR 1-2) (Figure 2). The distributions differed between users posting mostly negative tweets and users posting mostly neutral or positive tweets. Although there were more users posting neutral/positive tweets overall, the most prolific users during the time period were posting mostly negative opinions about HPV vaccines.

**Figure 3:**
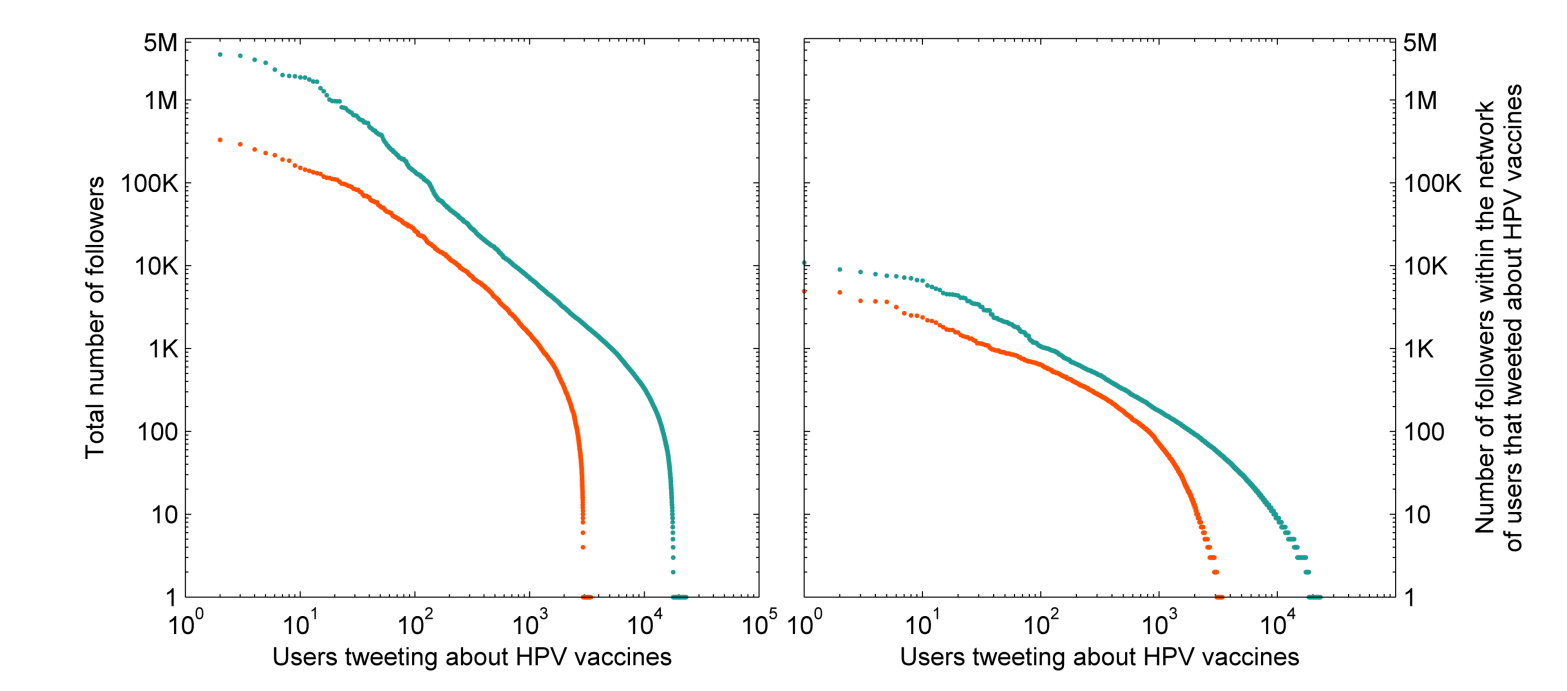
The ordered distribution of users according to the total follower counts (left) and follower counts within the network of 30,621 users (right). Each user is represented by a dot and colored by users that tweeted mostly negative tweets (orange) compared to all other users (cyan). The vertical axes are zero-adjusted to accommodate users that had zero followers.

We defined social connections as the sets of users that followed, or were followed by, the users that tweeted about HPV vaccines. The total number of unique followers for all users that tweeted about HPV vaccines in the 6-month period was 51,397,377. The total number of followers per user varied between 0 and 5,136,595 with a median of 274 followers per user (IQR 36-996) (Figure 3, left). Considering only the connections between users that tweeted about HPV vaccines, 957,865 social connections were identified and this defined the internal network of social connections among the 30,621 users. Followers per user in this internal network varied from 0 to 10,945 with a median of 8 followers per user (IQR 2-33) (Figure 3, right). Although news organizations and magazines made up the majority of users with the greatest number of followers overall, government health organizations and academic institutions or groups were more consistently featured among the set of users with the most followers in the internal network. Practitioners and writers (books and blogs) of specific forms of alternative medicine as well as antivaccine activists and celebrities did not feature among the set of users with the most followers overall, but occupied higher ranks when counting the number of followers in the internal network.

**Figure 4:**
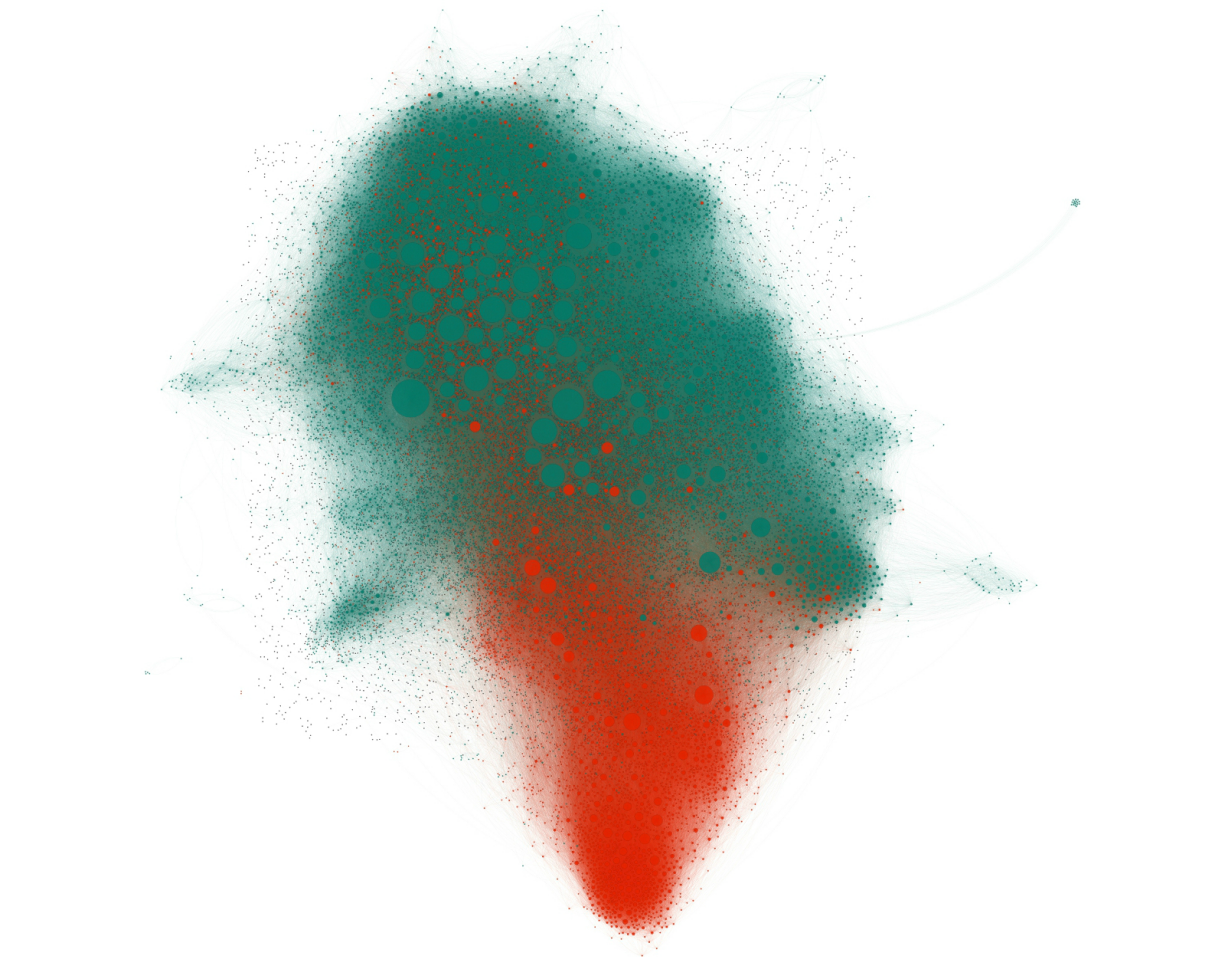
The network of 30,621 users that tweeted about HPV vaccines during the period between October 2013 and April 2014 organized via heuristic so that users are closer to other users with whom they are connected. The sizes of the nodes are proportional to the number of followers within the network. Users are colored according to information exposure (orange: those exposed to a majority of negative opinions; cyan: users that were exposed to mostly neutral/positive tweets; gray: users not exposed to HPV vaccine tweets).

Although only 25.13% (20,994/83,551) of tweets were classified as negative, 29.54% (9046/30,621) of users that tweeted about HPV vaccines appeared to be exposed more often to negative tweets than to neutral and positive tweets. This difference, and a visual interpretation of the network, suggests that users posting negative tweets about HPV vaccines were not evenly mixed in the network and often belonged to communities primarily consisting of users who also posted negative tweets about HPV vaccines (Figure 4).

Among the 30,621 users that tweeted about HPV vaccines, 18,657 users had timelines in which at least 1 tweet was posted after at least 3 exposures and were thus eligible for a temporal analysis of exposures and subsequent tweets. The likelihood of posting a negative tweet about HPV vaccines following a prior majority exposure to negative tweets was 37.78% (2780 of 7361 users). For users whose prior exposures were mostly neutral/positive, 10.92% (1234 of 11,296 users) subsequently posted a negative tweet. These results corresponded to a relative risk of 3.46 (95% CI 3.25-3.67, *P*<.001) indicating that users with greater prior exposure to negative opinions about HPV vaccines were more likely to express negative opinions.

To further test the association between exposure and expression within different groups of users, we undertook a post hoc subgroup analysis. Among the set of users that met the inclusion criteria and also had fewer than 1000 followers (n=11,845), we calculated the relative risk in the same way and found that the relative risk of posting a negative opinion about HPV vaccines after having been more often exposed to negative opinions about HPV vaccines was 3.61 (95% CI 3.32-3.93). For users with fewer than 500 followers (n=8790), the relative risk was 3.57 (95% CI 3.23-3.95) and for users with fewer than 300 followers (n=6521), the relative risk was 3.76 (95% CI 3.33-4.24). The results suggest that the association between previous exposure and subsequent expression was slightly stronger among Twitter users with fewer followers.

## Discussion

### Principal Findings

Approximately one-quarter of the tweets about HPV vaccines that were posted in the period were critical of the safety or value of HPV vaccines or actively encouraged vaccine refusal. These tweets, which included misinformation, anecdotes, and opinions that may result in vaccine hesitancy or refusal, made up the majority of HPV vaccine-related information exposures for nearly 30% of users that tweeted about HPV vaccines in the period. Our analysis of the network of follower relationships suggests that users expressing negative opinions about HPV vaccines tended to be more closely connected to users expressing the same opinions. Our analysis of the sequences of HPV-related tweets demonstrated an association between prior exposure to negative tweets about HPV vaccines and the subsequent posting of negative tweets about HPV vaccines. Together, these results suggest that homophily or contagion may play a role in the expression of negative opinions about HPV vaccines, but the study does not help to quantify their specific contributions [35].

To the best of our knowledge, ours is the first empirical study to consider the association between information exposure and subsequent expression for vaccines on social media. Other studies have used supervised machine learning to automatically classify tweets about vaccination [30] and the frequency of tweets over time exhibits a similar temporal pattern to the one we observed. Other studies have used Twitter as a laboratory to measure the propagation of negative news content, complaints, and rumors [36-38]. Other studies that considered misinformation were specifically aimed at differentiating between credible and not credible information, the containment of misinformation, and the identification of misinformation sources [39-41].

It is important to note that the study design we used precluded conclusions about what proportions of negative opinions expressed in the period were the consequence of exposure (contagion of opinions), the consequence of users creating connections to other users who already hold similar opinions (homophily), or if other external factors caused connected users to express similar opinions [35]. Alternative study designs that measure or model contagion from observable or synthetic networks are common in other application domains and more generally in network science [42-46], including where connections between nodes change over time [47-49].

Other studies have considered the news and online media representation of vaccines in different ways. One study examining the representation of vaccines in the media identified a rate of negative opinions in media reports for vaccines generally of 31% [33], with similar percentages in a study of US and Canadian news articles about HPV vaccines [50]. In comparison, 29% of US parents have reported being unsure about the vaccines for their children or otherwise delayed or refused vaccinations [51]. In the United Kingdom, very few newspaper articles (including tabloids) were classified as negative [52], whereas 19% of parents in England responded that they would not vaccinate their children in the future [53]. An Australian study found that HPV safety concerns were present in 39% of newspaper articles between 2006 and 2009 [19]. A study examining news media in the mid-1990s found that a small number of individuals were responsible for nearly half of all the statements opposing vaccination [54]. We found a similar pattern on Twitter for HPV vaccines using data from nearly 20 years later—where a small number of individuals posting negative opinions on Twitter produced a substantial proportion of the negative opinions. Given that these proportions are much higher than the average rates of vaccination refusal recorded in registries at approximately 2% [55,56], more work is needed to understand how population-level indicators of negative opinions might relate to vaccination decision making.

### Implications

Implications of this work include new avenues for understanding how community affiliation on Twitter corresponds to the exposure to misinformation, the subsequent expression of opinions, and individual decision making. The simple methods we used here may be of practical value for answering questions about how new information becomes established in different communities. For example, do the results of scientific studies demonstrating efficacy tend to spread primarily through scientific communities and not through communities of hesitant parents? Which popular news websites, influential users, or organizations are better connected to communities that are at higher risk of being exposed to, and subsequently affected by, misinformation? How often do young teenagers or their parents pass along negative opinions following encounters with misinformation or negative experiences with the vaccine process? Using new methods for classifying the location and characteristics of Twitter users [57,58], it may be possible to construct Twitter-derived indicators of skewed misinformation exposure in geographic areas and demographic strata, and these may be useful for predicting or reflecting localized shifts in decision making such as increases in refusal. From a practical perspective, this kind of information risk surveillance could be used to complement existing methods for gathering localized information (surveys, interviews, and registry analysis) and improve community engagement and public health actions by targeting resources more efficiently.

### Limitations

Limitations of this study come from our inability to track social connections as they appear and disappear during the period. Due to limits in the rates at which we access this information on Twitter, the social connections associated with each user were collected only once during the period, shortly after the first time we identified a relevant tweet by the user. However, by checking the consistency of connections between users within the set, we found that 81.6% of users’ connections were confirmed by the information from the other user (eg, a user’s follower is confirmed as someone the user follows), so we are reasonably confident that the connection structure was relatively consistent over time.

Our search terms were fixed and although we were careful to select search terms that covered the vast majority of the discussion about HPV vaccines without collecting irrelevant tweets, we may have missed a smaller number of tweets about the topic and these tweets may not have had the same proportion of negative opinions. Query-expansion techniques used to improve search strategies over time could be applied to address this limitation in future work [59-61]. Finally, we relied on an ensemble classifier rather than manual labeling, so a small proportion of the tweets will have been misclassified. However, the imperfections in the classifier are unlikely to have affected the results because the study was across large groups, our measure of exposure was based on counting the majority across a number of tweets rather than individual tweets, and the associations were clear.

### Conclusions

We found that Twitter users who were more often exposed to negative opinions about the safety and value of HPV vaccines were more likely to tweet negative opinions than users who were more often exposed to neutral or positive information. Although we were unable to determine the differential contributions of homophily, user characteristics, and contagion to this effect, the results provide a detailed view of negative opinions about HPV vaccines on Twitter in the period and indicate associations between the community structure, information exposure, and expression of negative opinions about vaccines among social media users. Ongoing surveillance of opinions about vaccination on social media may complement surveys and other public health surveillance methods to improve the efficiency and efficacy of public health communication strategies.

## Abbreviations

API: Application Programming Interface
HPV: human papillomavirus
IQR: interquartile range
